# One chance to get it right: improving clinical handovers for better symptom control at the end of life

**DOI:** 10.1136/bmjoq-2021-001436

**Published:** 2021-09-29

**Authors:** Gabriel Goldraij, Vilma Adriana Tripodoro, Melisa Aloisio, Sandra Analía Castro, Christina Gerlach, Catriona Rachel Mayland, Dagny Faksvåg Haugen, The ERANet-LAC CODE project group, Dagny Faksvåg Haugen

**Affiliations:** 1 Internal Medicine/Palliative Care Program, Hospital Privado Universitario de Córdoba, Córdoba, Argentina; 2 Instituto Universitario de Ciencias Biomédicas de Córdoba (IUCBC), Córdoba, Argentina; 3 Department of Palliative Care, Instituto de Investigaciones Medicas Alfredo Lanari, Buenos Aires, Argentina; 4 Institute Pallium Latinoamérica, Buenos Aires, Argentina; 5 Palliative Care Program, Hospital Privado Universitario de Córdoba, Córdoba, Argentina; 6 Interdisciplinary Palliative Care Unit, University Medical Centre of the Johannes Gutenberg University Mainz, Mainz, Germany; 7 Department of Palliative Care, Heidelberg University Hospital, Heidelberg, Germany; 8 Department of Oncology and Metabolism, The University of Sheffield, Sheffield, UK; 9 Palliative Care Institute, University of Liverpool, Liverpool, UK; 10 Regional Centre of Excellence for Palliative Care, Western Norway, Haukeland University Hospital, Bergen, Norway; 11 Department of Clinical Medicine K1, University of Bergen, Bergen, Norway

**Keywords:** palliative care, pain management, patient safety, quality improvement

## Abstract

Poor communication contributes to morbidity and mortality, not only in general medical care but also at the end oflife. This leads to issues relating to symptom control and quality of care. As part of an international project focused on bereaved relatives’ perceptions about quality of end-of-life care, we undertook a quality improvement (QI) project in a general hospital in Córdoba city, Argentina.

By using two iterative QI cycles, we launched an educational process and introduced a clinical mnemonic tool, I-PASS, during ward handovers. The introduction of the handover tool was intended to improve out-of-hours care.

Our clinical outcome measure was ensuring comfort in at least 60% of dying patients, as perceived by family carers, during night shifts in an oncology ward during the project period (March–May 2019). As process-based measures, we selected the proportion of staff completing the I-PASS course (target 60%) and using I-PASS in at least 60% of handovers. Participatory action research was the chosen method.

During the study period, 13/16 dying patients were included. We received 23 reports from family carers about the level of patient comfort during the previous night.

Sixty-five per cent of healthcare professionals completed the I-PASS training. The percentage of completed handovers increased from 60% in the first Plan-Do-Study-Act (PDSA) cycle to 68% in the second one.

The proportion of positive reports about patient comfort increased from 63% (end of the first PDSA cycle) to 87% (last iterative analysis after 3 months). Moreover, positive responses to ‘Did doctors and nurses do enough for the patient to be comfortable during the night?’ increased from 75% to 100% between the first and the second QI cycle.

In conclusion, we achieved the successful introduction and staff training for use of the I-PASS tool. This led to improved perceptions by family carers, about comfort for dying patients.

## Problem description

The provision of care for dying patients with cancer varies on a global basis.^1^ According to the Quality of Death Index and its corresponding indicators of quality of care at the end of life, Argentina ranks number 32 internationally and sixth on the American continent.^1^ The present work was part of the international ERANet-LAC CODE project (2017–2020).^2^ The project included an international survey, using the ‘Care Of the Dying Evaluation’ (CODE) questionnaire, focused on bereaved relatives’ perceptions about quality of end-of-life care in seven South-American and European countries.^3^ CODE is a 42-item, self-completion, post-bereavement questionnaire, focused on both quality of patient care and the level of family-carer support provided in the last days of life and immediate post-bereavement period.^3 4^ In Argentina, information and decision-making, support for relatives and environmental factors were the areas of care identified through the survey as needing to be improved.^2 3^

The Hospital Privado Universitario de Córdoba has 260 inpatient beds, 35 intensive care beds and 100 places for home care. The wards are staffed with teams in charge of the patients’ general care, but care is also received from specialist services each day. Oncology services include inpatient and outpatient consults, day care hospital, chemotherapy services and bone marrow transplantation services. The hospital is in the process of being evaluated to obtain a Joint Commission International (JCI) accreditation and certification.^5^ For the last 10 years, the hospital’s Specialist Palliative Care (SPC) programme has been working in several settings. The team is composed of four palliative care physicians, eight nurses, a social worker and a psychiatrist, who all provide mainly home-based palliative care services. Additionally, from the main team, two doctors, a social worker and a nurse work part time in outpatient clinics and as part of an inpatient advisory service. This team led the quality improvement (QI) project. Generally, the majority of the workload is in the home care setting (70%), with 30% spent in the hospital setting (inpatient and outpatient). The opportunity given by the QI project to improve care for the dying in the hospital was seen as a key priority for the SPC team.

Having received the results of the CODE survey, we conducted focus groups (FGs) with bereaved relatives from our hospital, sharing with them the survey results illustrating the quality of care for patients with cancer dying in hospitals. The FG participants further discussed the issues arising from the survey results. In particular, many participants stated that symptom control was often perceived to be inadequate at night, due to difficulty accessing ‘as needed’ analgesia and miscommunication between teams.

For example, one bereaved relative reported that, ‘One night, I went to ask doctors for extra doses of morphine because my husband was in pain, but doctors told me that they could not give me that medication because the oncology team was in charge of the patient, and at that moment it was not possible to communicate with them’. As a result, this patient was in pain until the next morning. We also received reports showing that important misconceptions about pain assessment and myths about morphine use were present among staff.

The input from the FG made our team realise that miscommunication about symptom relief was frequent at the interface between day and night shifts, and this became the starting point for our improvement process. We considered that using a clinical handover tool like I-PASS would improve the transfer of essential information about the patient’s care.^6^ We designed a QI project on an oncology ward at our teaching hospital.

Our clinical outcome measure was to ensure comfort for dying patients during night shifts on the oncology ward in at least 60% of cases during the project period March–May 2019. To verify the impact on the care for dying patients we would ask for family carers’ views.

The process measure was to use I-PASS in at least 60% of handovers. Before starting the project, no formal processes for handovers in end-of-life care had been used.

Our reporting of this QI project is in keeping with the Revised Standards for Quality Improvement Reporting Excellence (SQUIRE 2.0).^7^

### Background

End-of-life care involves a holistic approach to care, incorporating the relief of physical and emotional symptoms, effective communication among and between professionals and family members, and support for both the patients’ and relatives’ spiritual needs.^8 9^

Pain continues to be common at the end of life.^10^ Despite advances in understanding pain physiology and consensus on the effectiveness of available pharmacotherapies, many patients with terminal illnesses, such as cancer, report untreated pain.^11^

As pain relief is a fundamental human right, healthcare services are obliged to continually improve their pain management efforts. Furthermore, a Cochrane review suggested that policy makers have a duty to ensure that opioids are available for cancer pain relief.^11–13^

Good communication among patients, families and healthcare teams is crucial for effective end-of-life care, including pain control.^14^ Harms due to medical errors are an important cause of morbidity and mortality.^15^ In SPC settings, communication errors between professional teams are a frequent reason for discontinuity of care and the risk of previously controlled symptoms recurring, potentially causing unnecessary suffering at the end of life.^16 17^ Indeed, lack of effective communication, including miscommunication during patient care handovers from one healthcare professional to another, is a well-documented cause of ‘sentinel events’, the most serious that are reported to the JCI.^18^ JCI requires all healthcare providers to ‘implement a standardized approach to handoff communications, including an opportunity to ask and respond to questions’.^19^ The Accreditation Council for Graduate Medical Education also requires that residency programmes maintain formal educational programmes in handovers and care transitions.^20^ This requirement was already acknowledged by our hospital, and encouraged us to design the present project. We consider that high-quality care for dying patients should be integrated as a key aspect of inpatient oncological care 24 hours a day, 7 days a week.

It is imperative that institutional factors are considered in the context of a QI project. As specific individuals within the organisation can exert important influences (positive and negative), ensuring key stakeholder ‘buy in’ is essential to optimise the project’s chance of success.^21^

## Handover tool

**Figure 1:**
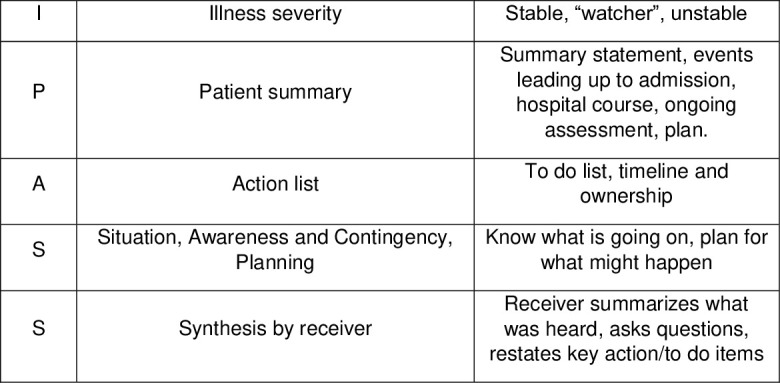
I-PASS mnemonic tool.

Handover is a real-time process of transferring patient-specific information from one caregiver or team to another.^22^ There are several mnemonic tools to improve handovers. At the time of starting the project, our hospital was already using one of them, namely I-PASS, a mnemonic to standardise verbal handover (figure 1).^6^ This instrument structures the clinical information in five areas, aiming to convey relevant information about the patient in a succinct and complete form and, at the same time, form a ‘shared mental model’ among all involved in the patient’s care.^23^ The use of I-PASS is reported to result in a reduction in the rate of preventable adverse events by 30%.^18^

In a recent study from the Netherlands, nurses providing palliative care reported that collaboration between care settings and information exchange were suboptimal.^24^ Adequate information handover was positively associated with improved timing and completeness of the information, and patients being well-informed about their disease and perspectives.

The IPASS tool was recently used in paediatric intensive care unit handovers in Argentina.^25^ A study showed that the intervention aided in the verbal and written transmission of critical information. All aspects of handover compliance were above 70%, with no changes in the time required to transfer the information for each patient.

### Measurement

The aim of our project was to improve symptom control by optimising handovers between care teams. Rather than ‘pain’ or ‘symptom control’, we chose ‘comfort’ as a global index of well-being in the last days of life. In Spanish, being ‘comfortable’ means overall well-being that includes not only having pain controlled, but also having a good night’s sleep, having clean bedsheets and so on. Hence, the word ‘comfort’ would be well understood in our population as an indicator of relief. Our operational definition of ‘comfort’ was the family carer’s perception about the patient′s overall well-being in the preceding 12 hours (the night), for patients perceived by a multidisciplinary team to be in the last hours or days of life. The second outcome variable ‘staff availability’ (to relieve symptoms) was also assessed by the subjective perception of the family carer.

The clinical outcome measure was ensuring comfort during night shifts for dying patients with cancer. This was assessed by the proportion of the patients’ relatives responding positively to two questions (taken from the CODE questionnaire) which were directly asked to the relatives who had been present during the night:

1. In your opinion, was the patient comfortable during the night?
2. In your opinion, did doctors and nurses do enough to keep the patient comfortable during the night?

A positive response to question 1 was ‘yes, all the time’; for question 2, the responses ‘yes, all the time’ and ‘it was not necessary, he/she was comfortable’ were deemed positive ones. Responses were recorded by members of the SPC team who acted as research staff.

As process-based measures, we selected:

1. The proportion of (involved) staff that completed the I-PASS course (target 60%).
2. The percentage of patient care handovers undertaken using I-PASS (target 60%).

Every time a patient was considered to be in the last days of life and the hospital’s integrated care plan for care of the dying was instituted, an SPC doctor met with the main ward team doctors to perform a handover using I-PASS. Researchers considered that the information transfer was adequately performed with I-PASS when all aspects were completed (oral report given and written document filled in). The following items were considered key for assessment, based on guidelines for observation of handoffs shaped from IPASS authors^26^: patient identification, illness severity, patient summary, action list, situation awareness and synthesis of receiver. However, the quality of the handover process was not assessed. Distractions or interruptions, together with the duration of each patient handover were not recorded.

As denominator, we counted all possible patient care handovers made during the included patients’ last days of life.

### Design

Based on the strengths of participatory action research (PAR), we designed our QI project as a ‘proof of concept’ idea.^21 27 28^ An important feature of PAR studies is acknowledging the ‘real world’ perspective generated by analysing data gathered from routine clinical practice.^21^ The role of the SPC team as participants as well as researchers in the PAR process contributed to the initial assessment and description during the project’s early stages. This is the point at which the end-of-life care scenario was evaluated through reflection before any modifications were made. Research led by service staff had a strong experiential basis.

**Figure 2:**
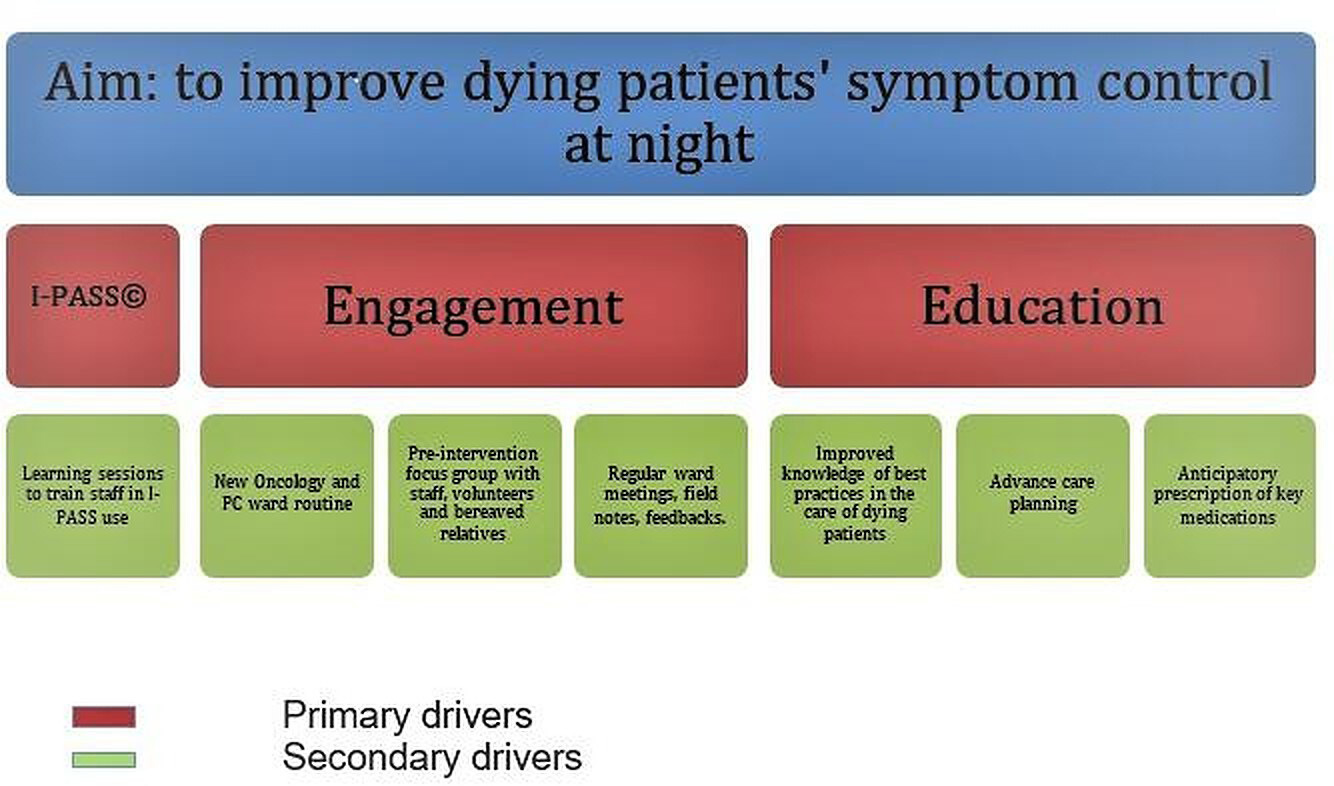
Quality improvement main drivers. PC, palliative care.

In phase 1, the preparatory phase, we considered three primary drivers deemed as essential to reach our main goal (figure 2). The first driver was the correct use of I-PASS during afternoon handovers to ensure essential medical information was conveyed to the night shift doctor. The doctors who met at the handover (the SPC doctors, oncologists, main ward doctors) would agree on an individualised care plan for each dying patient, including support for the family, and how to effectively provide symptom control.

We recognised that by using an instrument which was well aligned with hospital policies and promoted by the Patient Quality and Safety Department, this would be more readily accepted.

The second driver was to promote a strong commitment between key stakeholders including the SPC team, hospital physicians and nursing staff to introduce and adapt to the new process, recognising that sufficient ‘buy-in’ was essential. However, we realised the need for full involvement of the SPC team in the process, and consequently, as a team, we engaged fully throughout the communication process.

Finally, to provide timely care at night, an educational module was introduced to help enhance healthcare professionals’ skills in end-of-life care and symptom management, especially focusing on prescribing anticipatory ‘just in case’ medications and clarification of escalation plans.

### Strategy

We developed a plan of three phases and undertook two Plan-Do-Study-Act (PDSA) iterative cycles.

#### Phase 1: preparatory work. Relative and public involvement

Using purposive sampling, we conducted one FG with bereaved relatives and discussed the areas of improvement identified from the survey, in order to obtain new insights from their perceptions. Potential participants were identified from hospital records of recently deceased patients with cancer and their next-of-kin. Telephone contact was made at least 4–6 weeks after the patient’s death, inviting them to participate in the study. All the participants provided written informed consent prior to FG participation. The most assertive opinions presented in the FG were about symptom control, especially at night, as discussed earlier (in the Problem section).

Additionally, in a second FG, the perceptions of the SPC team, staff nurses and junior doctors enhanced our understanding of primary drivers. These identified themes—misconceptions about morphine use and lack of clarity about the patient’s dying phase needs—were included in our educational module. The goal of disseminating our end-of-life care plan throughout the institution prompted us to devote phase 1 to it.

With these outcomes, we attended an international research team meeting. We discussed the participants’ perceptions and understandings of the identified problem, how to approach the problem, and suggestions for interventions to bring about change. Experienced stakeholders were consulted as ‘critical friends’, as well as volunteers and bereaved relatives reviewing the project plan.

#### Phase 2: facilitating change phase

##### PDSA#1

We launched an educational module with different topics in its curriculum: recognising dying, use of I-PASS, and symptom control in the last days of life. The intervention consisted of providing training on the I-PASS attributes (figure 1) through two live sessions (workshops), and an online course with multiple-choice evaluation. Frequent feedback about their performance was given during sessions orally. Each session lasted about 90 min. The quality and patient safety department was actively involved in those workshops providing guidance and educational material. Additionally, a specific workshop focused on end-of-life care, pain and symptom management was arranged for junior and senior medical staff.

Each day that a dying patient’s care was being supported by our end-of-life care plan, the SPC team met with the ward team to ensure a good handover using I-PASS. Usually, the handover took place at 16:00, when the main team transferred the care responsibility to the team that was in charge at night. At least five doctors were involved in the handover, which was conducted in a room exclusive for doctors. Nurses were not involved in this process step, because of differences in their scheduled shifts.

The next morning, between 08:00 and 10:00, the relatives’ perceptions about the patient’s comfort level during the preceding 12 hours were collected by the SPC team, asking the specific questions described above. In addition, during the daily routine ward rounds, we took the opportunity for clinical discussion with the other teams to maintain reflection and awareness of the patient’s overall comfort. For example, discussions were prompted about the appropriateness of checking routine observations at night and the impact on patient comfort, and ways to obtain rapid access to spiritual counsellors. We realised that some communication difficulties were frequent at weekends, prompting the introduction of corrective measures. These measures marked the end of the PDSA cycle #1.

##### PDSA#2

We started the second PDSA cycle 7 weeks after the first cycle commenced. As mentioned previously, we recognised that more limited face-to-face contact with the SPC team at weekends could result in less optimal handovers. Consequently, telephone advice and support were provided in this next cycle. The SPC team provided ongoing support to the ward teams through training activities, feedback about team performance and anticipatory planning and prescribing.

Due to different working hours, the SPC team was unable to meet with all the nurses, especially those working on night shifts. We recognised the importance of this, however, addressing it by advising the doctors to share the goals of care with the nurses early during night shifts, and to contact them for any required patient reviews.

#### Phase 3: evaluation phase

To assess the effectiveness of the interventions, we performed two FGs, one with family carers and another one with stakeholders (healthcare professionals including a chaplain involved in the care of dying patients). We discussed four thematic areas under categories established on previous international survey^3^ CODE free text analysis: communication, context/atmosphere, care performance and attitude. Qualitative analysis from the FGs was embedded as insights into a new improvement cycle as part of a larger project beyond the aim of this report. We also evaluated the proposed process-based indicators.

## Results

During the QI project period, 16 patients with cancer died. As the SPC team was not informed about three patients who died at the weekend, these three cases were excluded from the subsequent assessment. Patient care was supported by our integrated end-of-life care plan for an average of 2.2 days, adding up to 24 possible days for which our outcome measures could be assessed. We obtained 23 reports. Our clinical outcome measures were the family carers’ perceptions about the level of comfort and care during the previous night (table 1).

**Table 1:** Results of asking relatives about the patient’s comfort the morning after the patient was considered to be in the last hours or days of life

PDSA	First question* In your opinion, was the patient comfortable during the night?				Second question In your opinion, did doctors and nurses do enough to keep the patient comfortable during the night?					Reports (n)*
	No, not at all	Yes, sometimes	Yes, all the time	Positive answer	No, not at all	Yes, sometimes	Yes, all the time	It was not necessary, he/she was comfortable	Positive answer	
PDSA #1	0	3	5	63% (5/8)	0	2	5	1	75% (6/8)	8
PDSA #2	1	1	13	87% (13/15)	0	0	9	6	100% (15/15)	15
										23

*Absolute numbers.

PDSA, Plan-Do-Study-Act.

We conducted the first outcome analysis in the second month (20 April), showing 63% (5 out of 8) positive reports by family members for patient comfort. In the subsequent PDSA analysis (end of May), the frequency of positive reports had increased to 87% (13 out of 15). In total, we obtained 18 out of 23 possible reports showing good levels of care.

The second clinical outcome measure, about healthcare professionals’ efforts to ensure patient comfort, increased from 75% to 100% positive answers from the first to the second PDSA analysis.

As regards our process indicators, 47/73 (65%) healthcare professionals completed the I-PASS training (target 60%). The percentage of handovers completed (orally and written) by using the IPASS increased from 60% (6 out of 10) in the first PDSA cycle to 68% (15 out of 22) in the last one (target 60%).

## Lessons and limitations

This QI project demonstrates that family carers’ perceptions about the care for dying patients can be specifically used to help improve patient care. To the best of our knowledge, this study is the first to evaluate a training programme on the use of I-PASS for healthcare professionals providing care for dying patients with cancer. To improve learning and enhance the effectiveness of QI work, involvement and collaboration between both researchers and practitioners were required.^29^

Involving family members in the action planning was the first and most valuable experience to trigger the project. Their input gave us the opportunity to identify a relevant problem lending itself to be improved. As a ‘proof of concept’ study conducted in a single site (acute general hospital), the aim was to assess locally if this training innovation was acceptable and feasible to healthcare professionals, with the view to design more complex interventions in the future.

Although, as a research team, we did not have a baseline measurement of the magnitude of the problem, the main concerns identified (lack of communication between teams, lack of basic knowledge about end-of-life care, and fear of morphine) were a serious threat to achieve end-of-life care goals and as such were important to focus on within a QI project.

Despite the oncology team being supportive of the project, the full commitment of the SPC team was needed to drive forward the study. An important lesson emerged from this limitation: contributing to solving a hospital problem, that is, the quality of care for dying patients with cancer, strengthened the awareness and prominence of the SPC team within the hospital as a whole.

Some other points also deserve consideration. The researchers who conducted the project were also the doctors who took part in the handovers and collected the family carers’ feedback. This means that there are points in the process where some bias could have been introduced. The action researcher’s status as an ‘insider’ or ‘outsider’ is one of the most distinctive characteristics.^21^ A person who has a formal position in the study setting is classified as an ‘insider’. This was the situation of the SPC team. On the other hand, except as part of the action research study, an ‘outsider’ has no formal involvement in the setting. We did not look into whether I-PASS usage differed when the researchers were involved versus when they were not.

However, PAR is a change-oriented process, with less clear boundaries between researchers and the people targeted by the investigation than in more strictly defined research projects.^21^ Apart from acting ethically, there is no optimal way to cope with the challenges involved in the action researcher–participant connection. As a result, action researchers must be aware of the potential for the future problems and take steps to anticipate and address them.

The main clinical outcome we set (the proportion of family carers’ positive reports about the care provided in the preceding 12 hours) is a proxy measure we adapted from the ‘CODE’ bereaved relative questionnaire. Nevertheless, it has not been formally validated as an indicator. The extreme frailty of dying patients almost always precludes obtaining a direct evaluation on their comfort. We asked these two questions in order to have a brief, feasible indicator from the family carers’ perspective. It was created to obtain the information during morning rounds in the dying patient’s room.

The controversy between ‘implementers and methodologists’ means that practitioners engaged in organisational improvement often can face challenges. However, many of them see local benefits from their work despite the lack of conclusiveness about the effectiveness of QI shown by research reviews.^28^ When it comes to long-term sustainability, our initiative would be an excellent opportunity to expand the end-of-life care programme beyond the cancer ward. Pertinent factors to be considered in the current context would include economic pressures caused by the COVID-19 pandemic, organisational concerns on the ward, with overlapping work tasks of different teams, and a shortage of personnel.

Due to our lack of baseline measures, we cannot conclude that our interventions solely led to improved outcomes. Our goal of better dying patient care at night is in fact a multicomponent intervention implying a cultural change process.^30^ The effectiveness of these PAR programmes is influenced by a variety of factors, including leadership, changing surroundings, implementation details and organisational history. According to Shahian, ‘all that is lacking is the will to implement’. Equally important as clinical trials are QI projects to implement research findings and improve healthcare delivery.

Improved patient care handovers and documentation as part of routine practice benefited healthcare professionals by providing important insights. In order to be able to further generalise our findings, we need to replicate our project on a bigger scale.

## Conclusion

Handovers are important moments in care transitions. These critical moments of time represent situations when miscommunication can occur, threatening the overall comfort and well-being for dying patients. Our study demonstrated that using education, PAR and involving relatives, healthcare professionals and researchers, it was possible to obtain the successful introduction of a clinical mnemonic tool (I-PASS) and training of involved staff in its use, leading to improved patient comfort and care, as perceived by family carers. Currently, I-PASS is being used in the general and oncology wards of our hospital. However, it is clear that a continuous education programme in palliative and end-of-life care for all healthcare professionals would be essential to sustain our achievements. Using the Quality of Death Index rankings as a benchmark and the collaborative approach adopted within QI activities could help drive further initiatives focused on improving and sustaining quality of care for dying patients in Argentina.^1^
